# Reply to Chen and Vitetta

**DOI:** 10.14309/ctg.0000000000000448

**Published:** 2022-01-13

**Authors:** Francesco Violi, Pasquale Pignatelli, Alessandra Oliva, Vittoria Cammisotto

**Affiliations:** 1 Sapienza University of Rome and Mediterranea Cardiocentro, Naples, Italy;; 2 Department of Clinical Internal, Anesthesiological, and Cardiovascular Sciences, Sapienza University of Rome, Itlaly;; 3 Department of Public Health and Infectious Diseases, Sapienza University of Rome, Rome, Italy;; 4 Department of General Surgery and Surgical Speciality Paride Stefanini, Sapienza University of Rome, Italy.

We thank Drs Chen and Vitetta for the interesting comment to our study regarding the detection of low-grade endotoxemia in patients with coronavirus disease-19 (COVID-19) (1,2). We found that in COVID-19 patients, lipopolysaccharides (LPS) was significantly higher than in controls (median values 50 vs 13 pg/mL, *P* < 0.001, respectively) and associated with an enhanced risk of venous and arterial thrombosis. The increase of circulating LPS may have different explanation, but enhanced gut permeability is likely to play a major role. Thus, patients with COVID-19 displayed a significant correlation between serum LPS and zonulin, an indirect marker of gut permeability (3). As outlined by Chen and Vitetta, commensal bacteria greatly contribute to gut barrier integrity, thereby changes of gut microbiota (dysbiosis) may affect gut permeability and enhance LPS translocation into systemic circulation. This hypothesis is consistent with experimental studies where influenza virus elicited gut dysbiosis (4) and with a study in patients with community acquired pneumonia, where we found enhanced circulating LPS with a significant correlation with zonulin (5). It seems that changes in gut permeability-mediated LPS translocation into systemic circulation is a common feature of lung infections. Systemic inflammation related to the underlying infection has been suggested to play a role (4). However, an important caveat of these studies is the lack of direct evidence of enhanced gut permeability in patients with lung infections. Therefore, a direct methodology using, for instance, quantification of the urinary excretion of orally administered macromolecules, such as polyethylene glycol and disaccharides (lactulose or cellobiose) in combination with monosaccharides (mannitol or rhamnose) needs to be used to support the existence of such lung-gut axis. Altogether, these data indicate that low-grade endotoxemia is a common feature of lung infections, but further study is necessary to examine its impact on clinical outcomes in short- and long-term follow-up.

## CONFLICTS OF INTEREST

**Guarantor of the article**: Francesco Violi, MD.

**Specific author contributions**: F.V. conceptualized and wrote the draft. P.P., A.O., and V.C. revised and edited the article.

**Financial support:** None to report.

**Potential competing interests:** None to report.
